# Cellular Mechanisms of Skin Diseases

**DOI:** 10.3390/cells12060945

**Published:** 2023-03-20

**Authors:** Mohamad Goldust

**Affiliations:** Department of Dermatology, Yale University School of Medicine, New Haven, CT 06510, USA; mohamad.goldust@yale.edu

Skin plays an important role in protecting and enhancing health. Because it interfaces with the environment, it plays a key role in immunity and protects the body against pathogens. Although substantial research has been carried out to clarify the pathophysiology of skin diseases, much remains to be discovered regarding the cellular mechanisms of cutaneous disorders. In this context, the editors of the journal *Cells* decided to create a Special Issue, entitled “Cellular Mechanisms of Skin Diseases.” It encompasses six articles covering various aspects of skin structure [1,2,3,4,5,6].

This Special Issue highlights various aspects of the skin, including, but not limited to, cutaneous structure and the pathophysiology of skin disorders. To be brief, the key published articles in this Special Issue addressed the following topics: First, atopic dermatitis and its environment [1,2]. Blicharz and colleagues stated that patients with atopic dermatitis have a high prevalence of Staphylococcus aureus encoding superantigens on lesional skin, nonlesional skin, and the anterior nares [1]. Brewer et al. demonstrated that keratinocyte differentiation in tandem with an inflammatory milieu (IL-4/13) or barrier disruption (TJDP treatment) substantially changes the susceptibility to viral infection [2]. A possible cellular mechanism of human hypertrophic scars and keloids was highlighted by Petrou and colleagues [3], and a 3D psoriatic skin model enriched in T cells and its gene profiling was shown by Rioux et al. [4]. Second, Nowicka and colleagues assessed various aspects of lymphomatoid papulosis (LyP) and mentioned that because of the expanded risk of lymphoma development, patients diagnosed with LyP need lifelong follow-up, and many of them will develop malignant neoplasms in the future [5]. Finally, the role of serum Th1, Th2, and Th17 cytokines in alopecia areata was stated by Szczepańska and co-authors [6].

Special thanks go to esteemed contributors from around the world who enriched this Issue with their valuable and interesting studies.
